# Acute superior vena cava collapse after thymic carcinoma resection and failed pericardial bypass rescued by endovascular stenting: a case report

**DOI:** 10.1186/s12893-026-03713-6

**Published:** 2026-04-06

**Authors:** Liangwei Yang, Chong Zhang, Enkuo Zheng, Weidi Zhao, Guofang Zhao

**Affiliations:** Department of Thoracic Surgery, Ningbo No.2 Hospital, Wenzhou Medical University, Ningbo, China

**Keywords:** Thymic carcinoma, Superior vena cava syndrome, Endovascular stenting, Autologous pericardial transplantation

## Abstract

**Background:**

Thymic carcinoma with superior vena cava (SVC) invasion is challenging. Intraoperative acute SVC syndrome may result from extrinsic compression after venous reconstruction. Intraoperative acute superior vena cava collapse due to extrinsic compression following thymic carcinoma resection and venous reconstruction appears to be infrequently reported and offers important insights into graft selection and rescue strategies.

**Case presentation:**

A 77-year-old woman with stage III thymic squamous cell carcinoma achieved partial response after cisplatin/nab-paclitaxel with camrelizumab. Resection included stapled SVC venoplasty; during chest closure, central venous pressure (CVP) surged to 40 mmHg. An autologous pericardial bypass between the right innominate vein and right atrium temporarily normalized CVP, yet a second CVP spike occurred after re-closure. Angiography revealed severe proximal SVC narrowing consistent with extrinsic compression. Immediate endovascular stenting with sequential balloon dilations restored patency and stability.

**Conclusions:**

When autologous pericardial bypass collapses under extrinsic compression, immediate endovascular stenting provides a rational, time-critical rescue to re-establish SVC flow. Recognizing mechanical causes of collapse and anticipating external pressure can guide graft selection and intraoperative decision-making.

## Background

Thymic carcinoma is a rare anterior mediastinal malignancy with a propensity for local vascular invasion and inferior outcomes compared with thymoma [1]. Radical resection with negative margins remains the mainstay of therapy, but when the superior vena cava (SVC) is involved, achieving oncologic clearance while preserving venous return is particularly challenging. Intraoperative acute SVC syndrome due to extrinsic compression after thymic carcinoma resection and venous reconstruction appears to be only occasionally described in the literature, and clear guidance for intraoperative surgical decision-making remains limited. We report a case of acute SVC obstruction after stapled venoplasty and subsequent autologous pericardial bypass that was ultimately rescued by immediate endovascular stenting, and we discuss mechanistic causes and technical lessons to inform graft selection and intraoperative management in similar emergencies.

## Case presentation

**Fig. 1:**
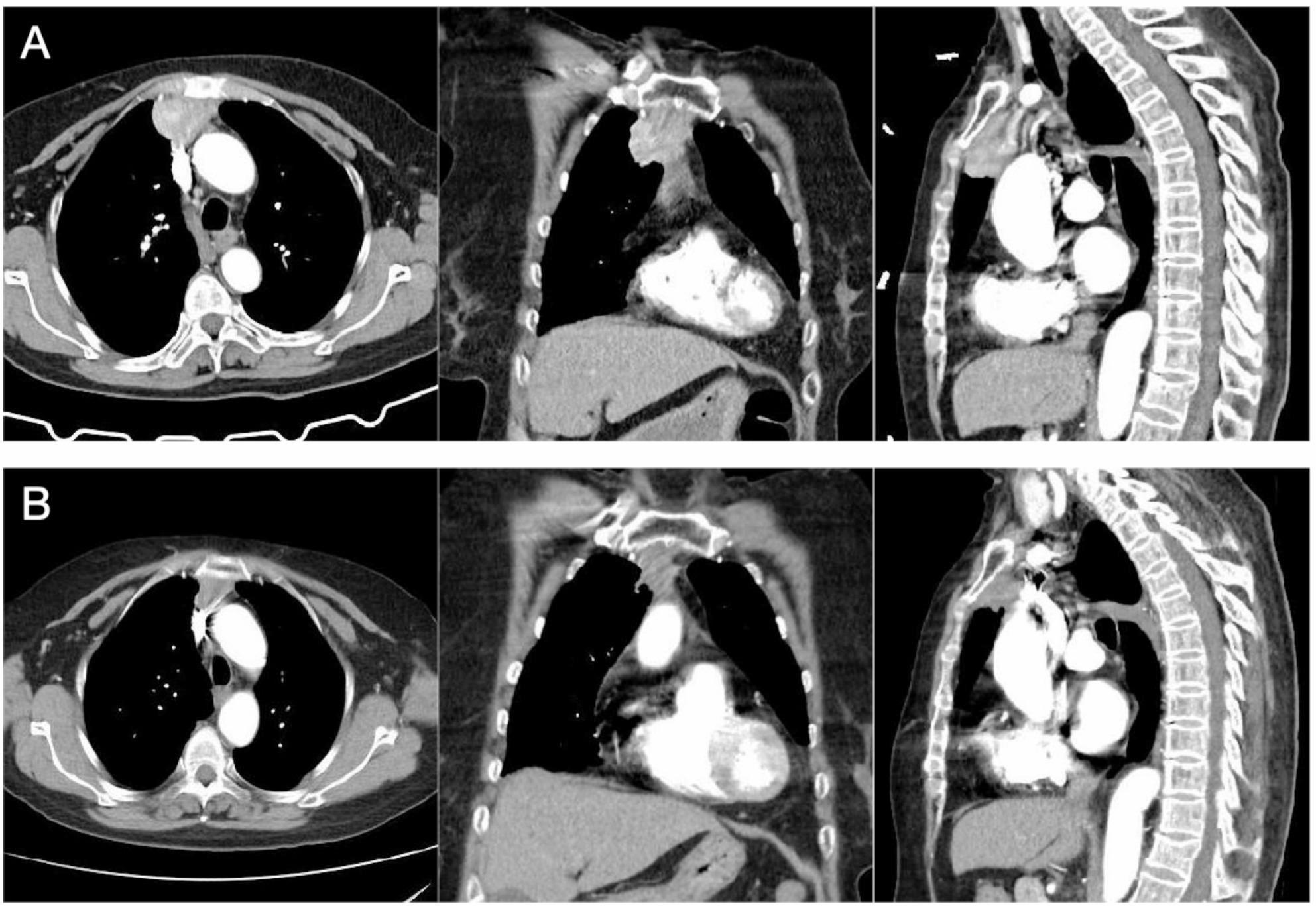
Pre- and post-neoadjuvant CT of thymic carcinoma invading the SVC. **A** Pre-treatment contrast-enhanced CT showing a lobulated anterior mediastinal mass invading the SVC and right upper lobe; **B** Post-neoadjuvant CT demonstrating partial response with tumor shrinkage

A 77-year-old woman presented with progressive chest wall venous distension. Contrast-enhanced computed tomography (CT) identified a 40 × 29 mm lobulated anterior mediastinal mass inseparable from the SVC and right upper lobe, with heterogeneous enhancement (pre-contrast 45 HU, post-contrast 136 HU, Fig. 1A). PET-CT showed avid uptake (SUVmax 18.6) and mildly avid mediastinal nodes (SUVmax 5.5). Serum CA-125 was 174.8 U/mL. Ultrasound-guided biopsy revealed squamous cell carcinoma consistent with thymic origin; clinical staging was cT3N1M0, Masaoka stage III.

Neoadjuvant therapy consisted of cisplatin 100 mg and nab-paclitaxel 300 mg with camrelizumab 200 mg every 3 weeks. After a grade 3 immune-related dermatitis following cycle one, immunotherapy was discontinued and chemotherapy continued for two cycles. Restaging CT showed tumor shrinkage to 22×20 mm (partial response, Fig. 1B).

**Fig. 2:**
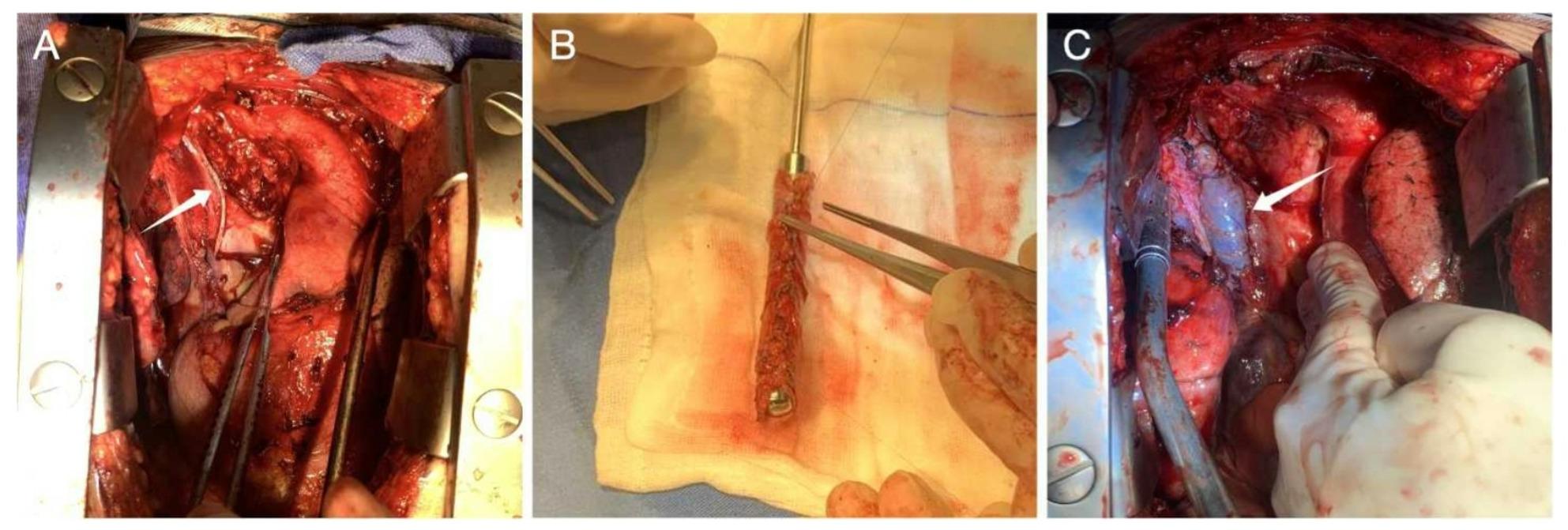
Intraoperative views of SVC reconstruction and autologous pericardial bypass. **A** Stapled partial resection of the superior vena cava (SVC) with venoplasty; the arrow indicates the staple line and the residual SVC lumen. **B** Preparation of an autologous pericardial tube graft (approximately 1 cm in diameter) for bypass reconstruction. **C** Completed bypass between the right innominate vein and the right atrium; the arrow indicates the pericardial conduit, which appears well-filled after restoration of venous flow

On May 6, 2024, median sternotomy was performed. The tumor infiltrated the SVC above the azygos arch and invaded the right upper lobe. The left innominate vein was not visualized, likely due to prior compression/occlusion. Because the tumor involved only a limited portion of the SVC wall, partial resection with stapled venoplasty was considered feasible. Before firing the stapler, the SVC was temporarily clamped and central venous pressure (CVP) was assessed to ensure that the residual lumen would maintain adequate venous return. A stapled partial resection of the SVC (~3 cm) was then performed using a linear stapler, leaving a luminal diameter of ~6 mm. CVP initially remained at 8 mmHg (Fig. 2A). During chest closure, CVP abruptly rose to 40 mmHg, prompting immediate reopening. A 1×9 cm tubular conduit was fashioned from autologous pericardium with 5-0 polypropylene and anastomosed between the right innominate vein and the right atrium (Figure 2B-C). CVP normalized to 8 mmHg; however, after re-closure, CVP again increased to 40 mmHg.

**Fig. 3:**
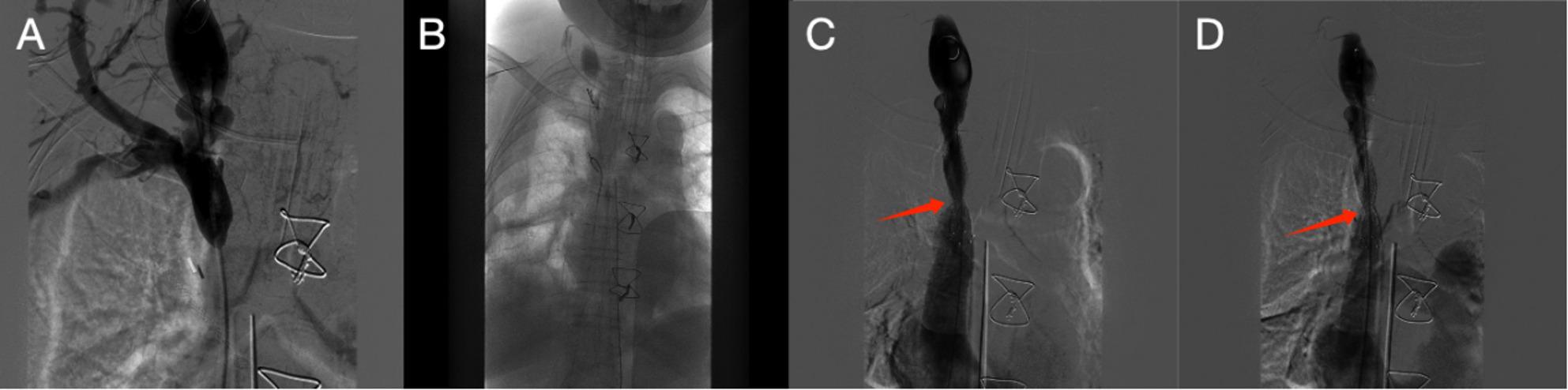
Intraoperative venography and endovascular rescue for acute SVC obstruction. **A** Emergency venography via bilateral femoral access demonstrating severe proximal SVC stenosis with pre-stenotic dilation and contrast stagnation. **B** Fluoroscopic view during deployment of a 10×80 mm self-expanding nitinol stent (Cordis) across the stenotic segment. **C** Post-stent venography showing residual focal narrowing at the proximal SVC due to extrinsic compression (arrow). **D** Final venography after sequential balloon dilations (6×80 mm and 8×60 mm) showing improved SVC caliber with restoration of luminal patency (arrow)

Emergency venography via bilateral femoral access demonstrated severe proximal superior vena cava (SVC) stenosis with pre-stenotic dilation and contrast stagnation, consistent with extrinsic compression rather than intraluminal obstruction (Fig. 3A). A 10 × 80 mm self-expanding nitinol stent (Cordis, USA) was deployed across the stenotic segment (Fig. 3B). Post-stent venography showed residual focal narrowing due to extrinsic compression (Fig. 3C), which resolved after sequential balloon dilations (6 × 80 mm and 8 × 60 mm), resulting in a widely patent lumen without contrast stagnation (Fig. 3D). After the final dilation, hemodynamics stabilized and CVP returned to baseline.

Postoperative management included low molecular weight heparin 4,000 IU once daily (subcutaneous) starting on postoperative day 1. No concurrent antiplatelet therapy was used. Chest drainage remained consistently acceptable without bleeding events. Chest-wall venous distension gradually resolved within one month. Pathology confirmed invasive squamous cell carcinoma of thymic origin; immunohistochemistry: CK5/6(+), P40(+), CD5(+), CD117(+), Ki-67 50%, TTF-1(-), TdT(-). At the 6-month follow-up at a local hospital, contrast-enhanced chest CT demonstrated no tumor recurrence and a patent superior vena cava without evidence of restenosis on venous phases. The CT images were reviewed by our team. Clinically, no recurrence of anterior chest-wall venous engorgement was observed. An intraoperative timeline of key hemodynamic changes and interventions is summarized in Table 1.

**Table 1:** Operative timeline and hemodynamics

Timepoint	Procedure/Event	CVP (mmHg)	Imaging/Findings	Action	Immediate outcome
After stapled venoplasty, before closure	Stapled partial SVC resection + venoplasty	8	Adequate return by inspection	Proceed to closure	Stable
During first closure	Chest closure	40	—	Immediate reopening	Suspected acute SVC obstruction
After reopening	Autologous pericardial bypass (R innominate → RA)	8	Good graft filling	Re-closure	Stable
During second closure	Chest re-closure	40	—	Emergency angiography	Recurrent obstruction
Intraoperative angiography	Bilateral femoral access	—	Severe proximal SVC narrowing with distal dilatation (extrinsic pattern)	Stent (10 × 80 mm) + balloon 6 × 80, 8 × 60	Patency restored
Post-procedure	ICU monitoring, POD1 LMWH 4,000 IU qd	8	No bleeding via drains	Continue anticoagulation per protocol	Hemodynamically stable
Follow-up (6 months)	Local-hospital contrast-enhanced chest CT	—	No recurrence; satisfactory SVC opacification	Routine surveillance	No recurrence of chest-wall varicosities

*CVP* central venous pressure, *RA* right atrium, *LMWH* low molecular weight heparin, *SVC* superior vena cava, *ePTFE* expanded polytetrafluoroethylene, *POD* postoperative day

## Discussion and conclusions

Radical resection of thymic carcinoma with SVC involvement is technically challenging. Surgeons must balance oncologic clearance against the risk of impaired venous return and acute hemodynamic collapse. Several reconstructive options have been described, including patch venoplasty, autologous pericardial bypass and prosthetic grafts [2–4], but intraoperative acute SVC syndrome due to extrinsic compression after reconstruction appears to be infrequently reported, and practical guidance in this setting is limited.

In the present case, the first episode of acute SVC obstruction occurred during chest closure after stapled partial SVC resection and venoplasty. A sudden rise in central venous pressure (CVP) to 40 mmHg at sternal approximation, without obvious kinking or thrombosis, suggested dynamic extrinsic compression within a confined anterior mediastinal space. A reconstructed SVC that appears patent with an open chest may collapse once the sternum is closed and intrathoracic geometry changes. Short, compliant venoplasty segments lying directly beneath the sternum or adjacent to pericardial reflections are particularly vulnerable, underscoring the need to consider the surrounding mechanical environment, not only luminal diameter, when planning reconstruction.

The second episode occurred after conversion to an autologous pericardial bypass between the right innominate vein and the right atrium. Autologous pericardium offers good biocompatibility and low infection risk and is widely used for SVC reconstruction [4],. In the present case, it was selected because the patient developed acute intraoperative SVC syndrome requiring urgent restoration of venous return, and autologous pericardium could be harvested rapidly from the operative field without additional incisions. In addition, the use of autologous tissue may reduce infection risk and is more economical compared with prosthetic grafts. However, its thin, highly compliant wall can collapse under external pressure even when anastomoses are technically sound. In our patient, the pericardial conduit restored flow while the chest remained open, yet obstruction recurred immediately at re-closure. In high-risk situations—such as bulky anterior mediastinal tumors, a narrow thoracic inlet and limited residual space after resection—ring-supported expanded polytetrafluoroethylene (ePTFE)、spiral saphenous vein graft or other stiffer prosthetic grafts may better resist external compression than autologous tubes.

The reconstructed venous segment, composed of native SVC and autologous pericardium, lacked rigid structural support and was therefore susceptible to external compression. After sternal closure, reduction of mediastinal space and surrounding tissue pressure may have resulted in dynamic narrowing of the reconstructed SVC segment.

This experience highlights the value of physiology-driven intraoperative monitoring and early multidisciplinary escalation. In both episodes, abrupt elevation of CVP to 40 mmHg with hemodynamic instability provided an immediate signal of SVC compromise. Continuous CVP monitoring is therefore essential in complex mediastinal resections with venous reconstruction, particularly during and after chest closure.

After failure of two open strategies, immediate endovascular stenting ultimately restored SVC patency, with maintained stent patency at the 6-month follow-up. Endovascular treatment is well established for malignant SVC syndrome in the palliative setting [5], but its use as an intraoperative or early postoperative rescue after open reconstruction is less frequently discussed. Our case illustrates that stent placement can provide a rapid, minimally invasive means to re-establish caval flow when a compliant graft or venoplasty segment collapses under external pressure. For centers with hybrid operating rooms or readily available interventional radiology, early involvement of the endovascular team should be considered in preoperative planning for high-risk anterior mediastinal tumors with major venous involvement.

Pre-existing venous collaterals may modulate the severity of acute SVC obstruction. In this patient, prominent chest wall collaterals on preoperative imaging likely provided partial decompression during periods of severe stenosis, potentially mitigating catastrophic cerebral or upper limb congestion. Although collateral formation cannot be influenced intraoperatively, preserving the azygos system and major tributaries whenever feasible may offer additional safety in the event of temporary or unexpected compromise of the reconstructed SVC.

Several practical considerations emerge from this case. First, preoperative imaging should be reviewed with attention to the anticipated mediastinal space after resection, to identify patients at risk of extrinsic compression after reconstruction and chest closure. Second, in such high-risk settings, conduit selection should account for wall stiffness and expected location, and ring-supported prosthetic grafts may be preferred for segments that will lie beneath the sternum or adjacent to an inflated lung. Third, continuous CVP monitoring and vigilance for abrupt changes at chest closure are critical for early recognition of SVC compromise. Finally, a predefined management pathway that includes prompt reopening of the sternum when needed and timely transition to endovascular stenting when extrinsic compression of a compliant reconstruction is suspected can shorten the time to definitive rescue.

In conclusion, intraoperative acute SVC syndrome during thymic carcinoma resection may result from dynamic extrinsic compression of a reconstructed segment at chest closure. Anticipating this mechanism, choosing conduits resistant to external pressure, and integrating rapid CVP-guided assessment with the option of immediate endovascular stenting may improve safety.

## Data Availability

All data generated or analysed during this study are included in this published article.

## Abbreviations

CT: Computed tomography
CVP: Central venous pressure
ePTFE: Expanded polytetrafluoroethylene
ICU: Intensive care unit
LMWH: Low molecular weight heparin
PET-CT: Positron emission tomography–computed tomography
SVC: Superior vena cava
WHO: World health organization
